# Prophylaxis, or Prevention of Dental Decay

**Published:** 1880-11

**Authors:** C. C. Patrick

**Affiliations:** Charleston, S. C.


### ARTICLE V.

### *Prophylaxis, or Prevention of Dental Decay.*

BY C. C. PATRICK, D. D. S., OF CHARLESTON, S. C.

[Read at N. Y. Meeting of Southern Dental Association.]

The efforts of those in charge of the Dental organs have been, from time immemorial, directed more towards limiting of the ravages of decay after its appearance than the adoption of a thorough and systematic method of *preserving* the teeth as their great Creator intended they should be—beautiful and perfect in their *simplicity*, unmarred by the art of the skillful Dental Surgeon.

Man (and every part of him,) was created perfect, and as long as he followed the laws and partook only of the good things Nature provided, his several organs performed their functions with such simple regularity that the diseases which now afflict us were almost unknown. "Men," of course, "died and people buried them," but "hardly ever" of syphilis, consumption, scrofula, or diseases of the digestive apparatus. The curse, "in pain shalt thou bring forth children," was intended to people the earth and to fill the grave yard. In all primitive communities where nature's laws have not been transgressed, the acts of parturition and teething caused as little anxiety and were fraught with as little danger to mankind as the brute creation. The savage, who, a half hour after giving birth would, with papoose on back, continue her march as if nothing had occurred, never dreamed of the obstetrician's forceps or

Cæsarian operations. The cutting of the teeth, which now brings grief and distress to almost every household in civilized countries, caused no fear in the simple societies of olden times, and does not seem to be fatal in the barbarous or rural tribes of modern days.

Though man in civilized countries of to day may not be more prone to destructive constitutional diseases, or have worse teeth than the luxurious Persians, Greeks and Romans of old, it is only in the frugal, nature loving country people of modern times that we can hope to find an approach to pristine excellence and perfection. The child of nature is always perfect; that of luxury and vice, a caricature. It is also too true that the "wages of sin is death," and few sins are greater than the wilful destruction of self or any portion of one's self. As surely as man transgresses the laws of nature in any age or any clime will he pay the penalty. As surely as he discards the most important and nutritious elements of food and then fixes up what remains in all manner of unrecognizable forms and bolts it without first putting the system, by exercise, in condition to assimilate it, will he destroy his originally perfect organism, bring disease and misery on himself and require the aid of Physician and Dentist. As sure as he gives unbridled license to his animal passions will he stamp his sin on millions unborn and bequeath to innocents destructive constitutional diseases that the brute creation know not of—diseases so destructive that it has been said, that could syphilis be stopped one half the diseases which now afflict mankind would disappear from the face of the earth. But as it is not probable that man will curb his desires or deny himself anything in an age where luxury, false notions and vice are the rule and not the exception, the effort to benefit him by showing him the error of his ways and leading him back to a proper mode of life, will be attended with enormous difficulties and successful only in as much as we receive her co operation. But that *should* not deter us as dentists and humanitarians from doing all in our power to build him up and give him good sound teeth. This we cannot do alone. The teeth are so intimately connected with the general system, sympathizing with, affecting and being influenced by all changes in it, that we could not hope to succeed in benefiting them by merely local attention, nor would the best constitutional treatment at this period make a perfect set of teeth. The health and strength or disease of them is implanted so early that it would be the height of folly to wait until birth to try to build a perfect structure on a weak or imperfect foundation. Treatment to be effective should be from the very beginning.

As *our* case begins with our *patient* in the *office*, and as all preventive measures should have taken place before this, it is our duty to associate with us those who have to stand at the helm and pilot him safely into the world. The Physician now recognizes the intelligent Dentist as a specialist of his noble profession, and is no longer averse to consult with or to give to his care cases which more strictly belong to the province of Dentistry.

There are here, gentlemen, no conflicting interests, no professional jealousies to excite, and the union can only result in the greatest good to mankind and be of incalculable assistance in the endeavor to preserve teeth from decay. How else could we hope to successfully combat the evil influence of disease or build up and restore to health weak and broken down constitutions. It is, therefore, incumbent on us to work faithfully and conscientiously together, and in order to have our efforts crowned with success we must commence our treatment with the woman in the earliest stages of pregnancy. We must frequently examine her mouth and see that all particles of tartar and other foreign bodies are removed, and the secretions and soft parts are in a normal condition. The greatest cleanliness should be enjoined and at the same time she should receive a most generous diet and a great deal of open air exercise. The food should abound in the bone and teeth producing phosphates. Should she be weak or delicate, tonics and the phosphates, or carb. phosphate of lime in cod liver oil or otherwise, should be administered, thereby preventing injury to the mother and assisting the little one in uteri. The same care regarding exercise, diet and attention to the oral cavity should be shown her after delivery. Should her milk be of any but the best quality it should be immediately stopped, and goats, cows, or still better, that of a healthy woman of sound parents, supplied. But as this class of beings is not over-moral, and prone to deceive, too much care cannot be exercised in the selection. No woman suffering from syphilis, consumption, scrofula, or any of the forms of insanity, should under any circumstances be allowed to nurse a child.

Should the offspring inherit a constitutional taint, or develop dyspepsia, a most thorough system of treatment should be instituted. When nature, by the eruption of the temporary teeth, denotes that a more solid and substantial food than milk is necessary, food with a large percentage of the phosphate of lime should be given ; such, for instance, as barley bread, oat meal, cracked wheat and unbolted flour. Too much stress cannot be laid on the avoidance of the abuse of candies and sweet-meats, for though they may be retained in the mouth without serious injury, they destroy the digestion and thereby the teeth. Fresh air, exercise and great bodily cleanliness are of a great importance to the young or the old. The child should be taught to brush its teeth as soon as it can handle a brush, and previous to this the mother or nurse should do it. The mouth should be frequently examined, particularly after illness of any kind, where acids or strong medicines have been used. All broken down, deciduous teeth and all impurities, injurious to the permanent, should be removed and the mouth rendered healthy. Crowded and irregular teeth should be filed, extracted, or brought to proper position in the arch as the judgment would dictate. And, in short, gentlemen, nothing should be left undone by Physician and Dentist to remove all causes, constitutional or local, favoring in the slightest degree decay.

